# Artificial intelligence-based model for diagnosing *Helicobacter pylori* in whole-slide images

**DOI:** 10.3389/fmed.2025.1594614

**Published:** 2025-06-11

**Authors:** Kehan Teng, Lihua Ren, Xiaoyu Yan, Yawei Duan, Zhe Chen, Hansheng Li, Lihua Zhang, Lei Cui

**Affiliations:** ^1^Department of Pathology, Zhongda Hospital, Southeast University School of Medicine, Nanjing, Jiangsu, China; ^2^Department of Gastroenterology, Zhongda Hospital, Southeast University School of Medicine, Nanjing, Jiangsu, China; ^3^School of Information Science and Technology, Northwest University, Xi’an, China

**Keywords:** *Helicobacter pylori*, gastric mucosal biopsy, pathological diagnosis, artificial intelligence, chronic gastritis

## Abstract

**Introduction:**

*Helicobacter pylori (H. pylori)* infection is considered to be a primary causative factor for gastric cancer and a common cause of chronic gastritis worldwide. Identifying *H. pylori* infection through hematoxylin and eosin (H&E) staining is demanding and tedious for pathologists. We aimed to use artificial intelligence (AI) models to improve the accuracy and efficiency of *H. pylori* diagnosis and to reduce the workload of pathologists.

**Methods:**

Here, we developed three multi-instance learning (MIL) models: AB-MIL, DS-MIL, and Trans-MIL, to automatically detect *H. pylori* infection. A total of 1,020 digitized histological whole-slide images (WSI) from 817 patients were used for training, validating and testing sets at a ratio of 3:1:1. Additionally, 100 cases (218 WSIs) were randomly selected from the test set for pathologists to identify *H. pylori* under the microscope. The accuracy, specificity, sensitivity, false negative rate, false positive rate, and other metrics were calculated separately for the MIL models and the pathologists.

**Results:**

All three models demonstrated good diagnostic performance in predicting *H. pylori* infection, with the DS-MIL classification model showing the best diagnostic performance, achieving an accuracy of 89.7% and an area under the curve (AUC) of 0.949, which is higher than the accuracy rate of senior pathologists at 81.7%. Furthermore, the model demonstrates superior performance in terms of sensitivity and specificity. The reliability of DS-MIL is confirmed through the Visual model.

**Discussion:**

Our research presents an AI - based predictive model for *H. pylori* infection, which significantly enhances clinical efficiency and diagnostic accuracy. Currently, we are conducting multi-center validation to enhance the model’s generalization capability.

## Introduction

*Helicobacter pylori* (*H. pylori*), a Gram-negative bacterium, is a well-established colonizer of the human gastric mucosa and a significant pathogen implicated in the etiology of various diseases. From 2011 to 2022, the global prevalence of *Helicobacter pylori* (Hp) was 43.1% (1). It is recognized as a major pathogenic factor in chronic active gastritis, peptic ulcers, gastric mucosa-associated lymphoid tissue (MALT) lymphoma, and gastric cancer. Gastric cancer associated with Hp infection accounted for 63.4% of all gastric cancers (2). Furthermore, it is associated with extra-gastrointestinal diseases such as cardiovascular, neurological, and immune disorders (3–5). Despite the substantial health risks posed by *H. pylori*, the clinical manifestations of infection are often subtle, with many infected individuals remaining asymptomatic or exhibiting non-specific symptoms.

Gastric mucosal biopsy is considered the gold standard for *H. pylori* detection. However, the microscale of the bacterium and the interference from gastric pit debris make its identification in hematoxylin and eosin (H&E)-stained sections challenging and labor-intensive for pathologists, leading to unsatisfactory sensitivity and specificity of diagnosis (6). In particular, post-treatment with proton pump inhibitors (PPIs) or other medications, *H. pylori* may undergo a spherical transformation, complicating its identification and increasing the risk of false-negative results (6, 7). Immunohistochemical (IHC) staining offers specificity, sensitivity, and accuracy rates exceeding 98%, unaffected by bacterial activity and spherical transformation (7), but it is more costly. Therefore, there is a pressing necessity for more precise, efficient, and cost-effective auxiliary techniques.

The available evidence suggests that *H. pylori* exerts a range of effects on gastric epithelial cells, leading to the impairment of epithelial barrier function, inflammation promotion, and oncogenic transformation (8, 9). Consequently, the presence of *H. pylori* infection can be inferred from certain morphological characteristics, including the presence of superficial (upper 1/3) band-like lymphoplasmacytic infiltration, intraepithelial acute inflammatory cell infiltration, varying degrees of damage to the gastric mucosal epithelium, and intestinal metaplasia (10). Recently, the application of artificial intelligence (AI) technology in the field of pathology diagnosis has garnered significant attention, encompassing tasks such as the segmentation of pathological areas, the localization and quantification of cells, and the prediction of immune phenotypes or molecular classifications (11–13). To the best of our knowledge, there are less studies applying AI for the detection of *H. pylori* infection on H&E-stained slides.

We hypothesized that an AI-based diagnostic model could be developed to automate the detection of *H. pylori*, leveraging state-of-the-art algorithms, advanced computing power, and big data. The objective of our study is to construct an AI model that analyzes morphological changes in *H. pylori*-positive H&E-stained sections, aiming to the presence of *H. pylori* infection. This approach aims to enhance diagnostic sensitivity and specificity while reducing the workload of pathologists.

## Methods

### Samples and assays

**Figure 1:**
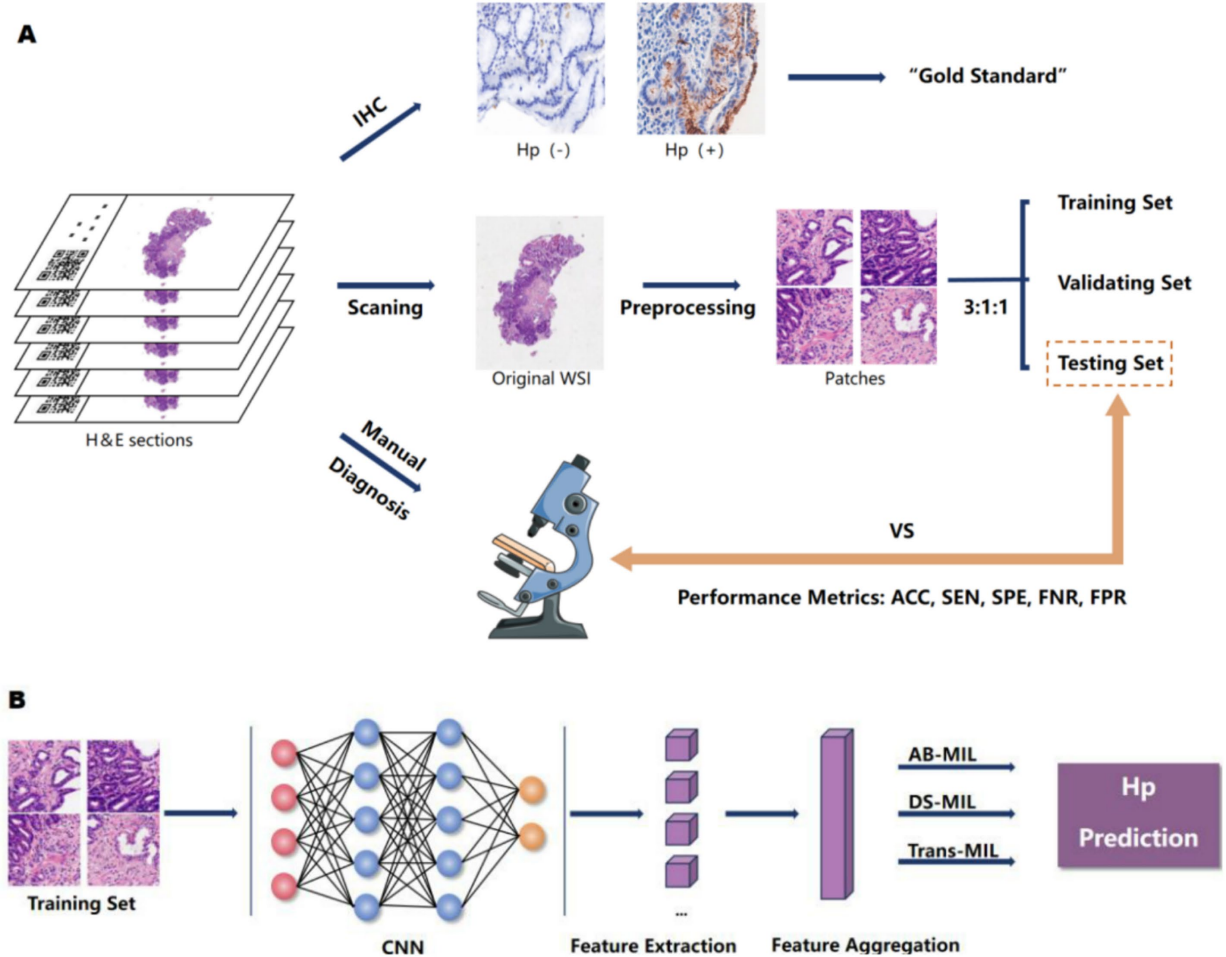
The research design concept. **(A)** We scanned H&E-stained sections from patients with chronic gastritis/chronic atrophic gastritis to obtain WSIs. All cases underwent *H. pylori* immunohistochemical staining, with IHC results serving as the gold standard. Cases were randomly divided into training, validation, and test sets at an 3:1:1 ratio. Pathologists also assessed *H. pylori* infection based on HE sections. We compared the diagnostic performance between the model and pathologists by calculating their accuracy, specificity, sensitivity, false negative rate, and false positive rate. **(B)** The neural network was trained on the training set, fine-tuned and optimized based on its performance in the validation set, and its overall efficacy was evaluated in the test set.

The gastrointestinal mucosal biopsy cases diagnosed as chronic gastritis/chronic atrophic gastritis were retrospectively collected in the Pathology Department of Zhongda Hospital affiliated with Southeast University from September 2022 to September 2023. Cases with poor quality that did not meet the research requirements were excluded, such as those with tissue section folding, knife marks, poor staining, discoloration, or too few specimens in the section affecting observation. *H. pylori* immunohistochemical staining was performed using *H. pylori* immunohistochemical working solution (purchased from Lan’ou Medical Technology Co., Ltd.) on the Dako fully automatic immunohistochemical staining machine, and the immunohistochemical results were used as the gold standard for *H. pylori* infection diagnosis. The IHC staining outcomes were interpreted by two pathologists. When there was a disagreement between the two pathologists, a third pathologist’s expertise was enlisted to provide an adjudication, ensuring the reliability and accuracy of the diagnostic assessments. *H. pylori* immunohistochemical positivity was defined as the presence of *H. pylori* infection. High-quality whole-slide images (WSI) were acquired using the Hamamatsu (Japan) and BingLi (China) whole-slide scanners at a 20x magnification, and the images were preprocessed to meet the training requirements of the deep learning model. Our research design is illustrated in Figure 1.

This study has been reviewed and approved by the Ethics Committee of Zhongda Hospital affiliated with Southeast University (2024ZDSYLL330-Y01).

### Algorithm

**Figure 2:**
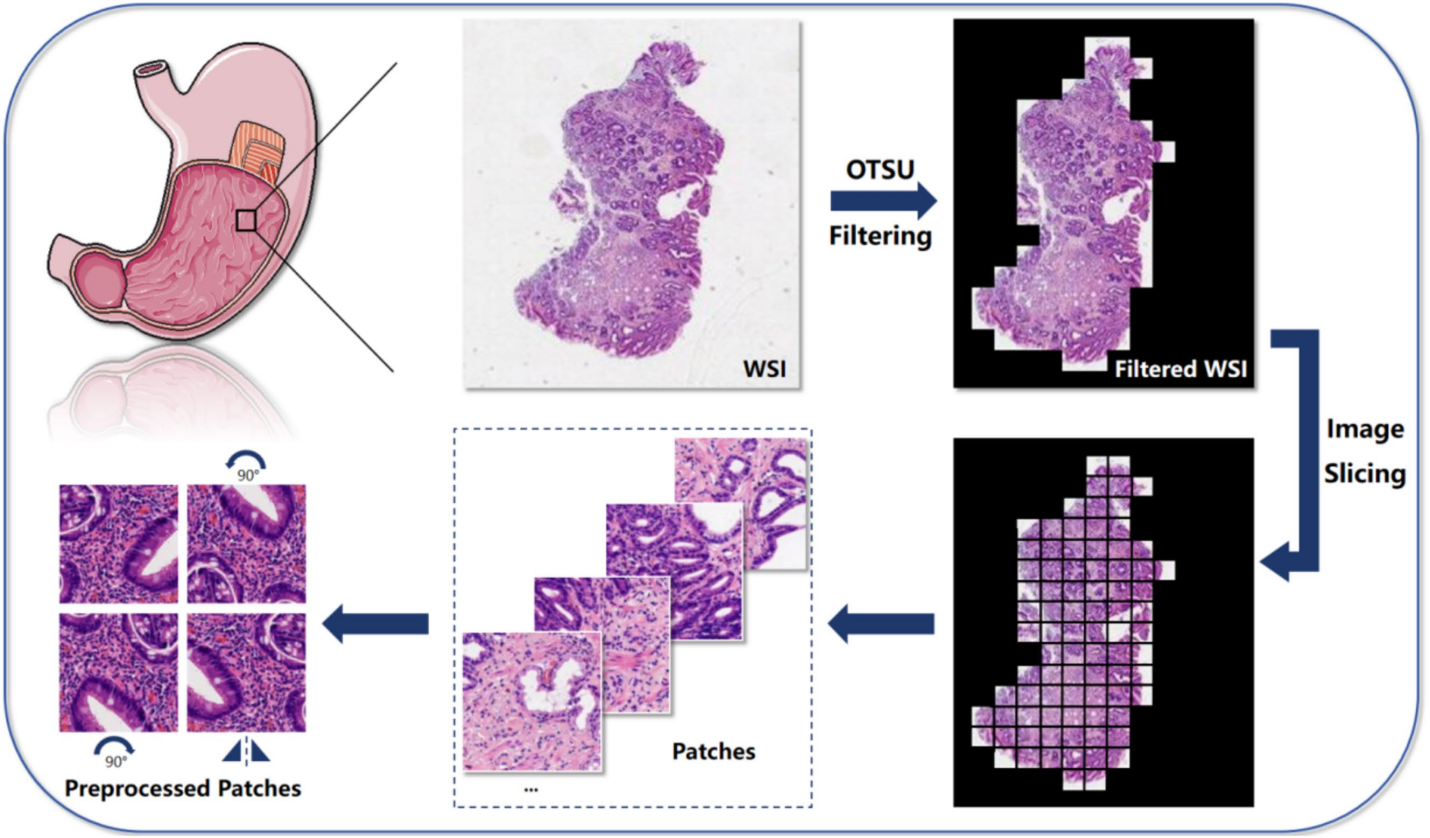
The algorithm is mainly divided into 5 steps: first, use the Otsu method to filter the background of the input Whole-slide image, highlighting the relevant tissue parts; then, slice the filtered image into small patches for further analysis at the local level; next, extract key features from these image patches for subsequent analysis and classification tasks; then, aggregate and combine the extracted features to form a comprehensive feature representation, containing information from all blocks; finally, we employed three MIL algorithms, which are AB-MIL, DS-MIL, and Trans-MIL, and compared the results to achieve the final predictions or classifications based on grouped instances.

This study utilized the *H. pylori* whole-slide imaging dataset, comprising a total of 1,020 images. The dataset was divided into training, validation, and testing sets in an 3:1:1 ratio, with the division based on individual whole-slide images (WSIs). Initially, the whole-slide images were segmented, with the background tissues filtered out using the Otsu method. These segmented images were then further divided into 256 × 256 pixel patches under 20× magnification. Figure 2 demonstrates the contrast between the whole-slide images before and after tissue background filtration, showing that the Otsu method effectively removes a significant portion of the background tissues, allowing the model to focus more on the tissue regions. Subsequently, the information from all patch images was embedded into a single feature, creating the feature file for the entire dataset. Feature extraction was performed using a ResNet50 model pretrained on ImageNet. Following this, the model was trained using the feature file from the initial dataset split, and the model’s performance was evaluated on the validation set at each epoch. Training was halted when the loss showed minimal improvement or began to increase. Finally, the best-performing model was used for testing. This study utilized three MIL methods, namely AB-MIL, DS-MIL, and Trans-MIL, for the classification of *H. pylori* positive and negative cases.

The core of using the AB-MIL (14) method for WSI classification lies in weighting each instance through the attention mechanism. This method multiplies the features of each instance by its corresponding attention score and aggregates the weighted features to obtain a representation of WSI, which is then classified by the classifier.

The DS-MIL (15) method employs a dual-stream architecture for WSI classification, which consists of two main streams. The first stream identifies key instances via max pooling. The second stream calculates the similarity of each instance to the key instances, generates attention weights, and aggregates the features. The final bag score is the average of the scores from both streams, which is used for the final WSI classification.

The Trans-MIL (16) method utilizes the self-attention mechanism of the Transformer to capture correlations between instances and encodes spatial information via the Pyramid Position Encoding Generator (PPEG) module for WSI classification.

### Evaluation metrics

The predictive evaluation metrics for the classification diagnostic model include Accuracy (ACC), Precision (PRE), Sensitivity (SEN), Specificity (SPE), False Negative Rate (FNR), False Positive Rate (FPR), and F1-score. These metrics are calculated from the true positives (TP), true negatives (TN), false positives (FP), and false negatives (FN) in the confusion matrix, with the following formulas. The receiver operating characteristic (ROC) curves are plotted for each model, and the area under the curve (AUC) is calculated.


$$\text{ACC}=\frac{\text{TP}+\text{TN}}{\text{TP}+\text{TN}+\text{FP}+\text{FN}};$$

$$\text{PRE}=\frac{\text{TP}}{\text{TP}+\text{FP}};$$

$$\text{SEN}=\frac{\text{TP}}{\text{TP}+\text{FN}};$$

$$\text{SPE}=\frac{\text{TN}}{\text{TN}+\text{FP}};$$

$$\text{FNR}=\frac{\text{FN}}{\text{TP}+\text{FN}};$$

$$\text{FPR}=\frac{\text{FP}}{\text{FP}+\text{TN}};$$

$$F1-\text{score}=\frac{2\times \text{PRE}\times \text{SEN}}{\text{PRE}+\text{SEN}}$$

### Manual diagnosis

A random selection of 100 cases (a total of 218 slides) from the test set was independently reviewed by a group of junior pathologists, a group of intermediate pathologists, and a group of senior pathologists (2–4 pathologists in each group), none of whom were aware of the immunohistochemistry results. The pathologists with fewer than 5 years of professional experience are categorized as Junior, those with 5 to 10 years of experience are classified as Intermediate, and those who have dedicated over 10 years to the study of gastrointestinal pathology are designated as Senior. The ACC, SPE, SEN, FNA, and FPR of the diagnosis made by the junior, intermediate, and senior pathologists were calculated, respectively.

### Statistical analysis

Statistical analysis was conducted using SPSS 27.0 software. The Shapiro–Wilk test was utilized to assess whether the metrics of each model adhere to a normal distribution. If the data conform to a normal distribution, an Analysis of Variance (ANOVA) test is applied; if the data do not conform to a normal distribution, the Kruskal–Wallis test is employed to examine the differences between the models. Additionally, we used the chi-squared (*χ*^2^) test to assess the differences in diagnostic performance among pathologists of various levels, as well as between the DS-MIL model and pathologists. A *p*-value < 0.05 was considered statistically significant.

## Results

### Case numbers and characteristics

In this study, we collected cases diagnosed with chronic gastritis/atrophic gastritis from the Department of Pathology at Zhongda Hospital affiliated with Southeast University between September 2022 and September 2023. A total of 817 cases, comprising 1,020 histological images were enrolled, with 436 cases (530 images) identified as *H. pylori*-positive and 381 cases (490 images) as *H. pylori*-negative. These cases were divided into training, validation, and testing sets in a ratio of 3:1:1. The evaluation metrics for the models included accuracy, sensitivity, specificity, and others.

### Performance metrics of three models: AB-MIL, DS-MIL, and Trans-MIL

Here, we developed three multi-instance learning (MIL) models: AB-MIL, DS-MIL, and Trans-MIL to test the prediction of *H. pylori* infection. The AB-MIL (Attention-Based Multiple Instance Learning) is an attention-based multi-instance learning framework that assigns importance scores to instances within a bag. The DS-MIL (Dual-Stream Multiple Instance Learning) is a dual-stream architecture for WSI classification that integrates self-supervised contrastive learning with a novel MIL aggregator. The Trans-MIL is a Transformer-based methodology tailored for correlated MIL in the context of WSI classification.

**Figure 3:**
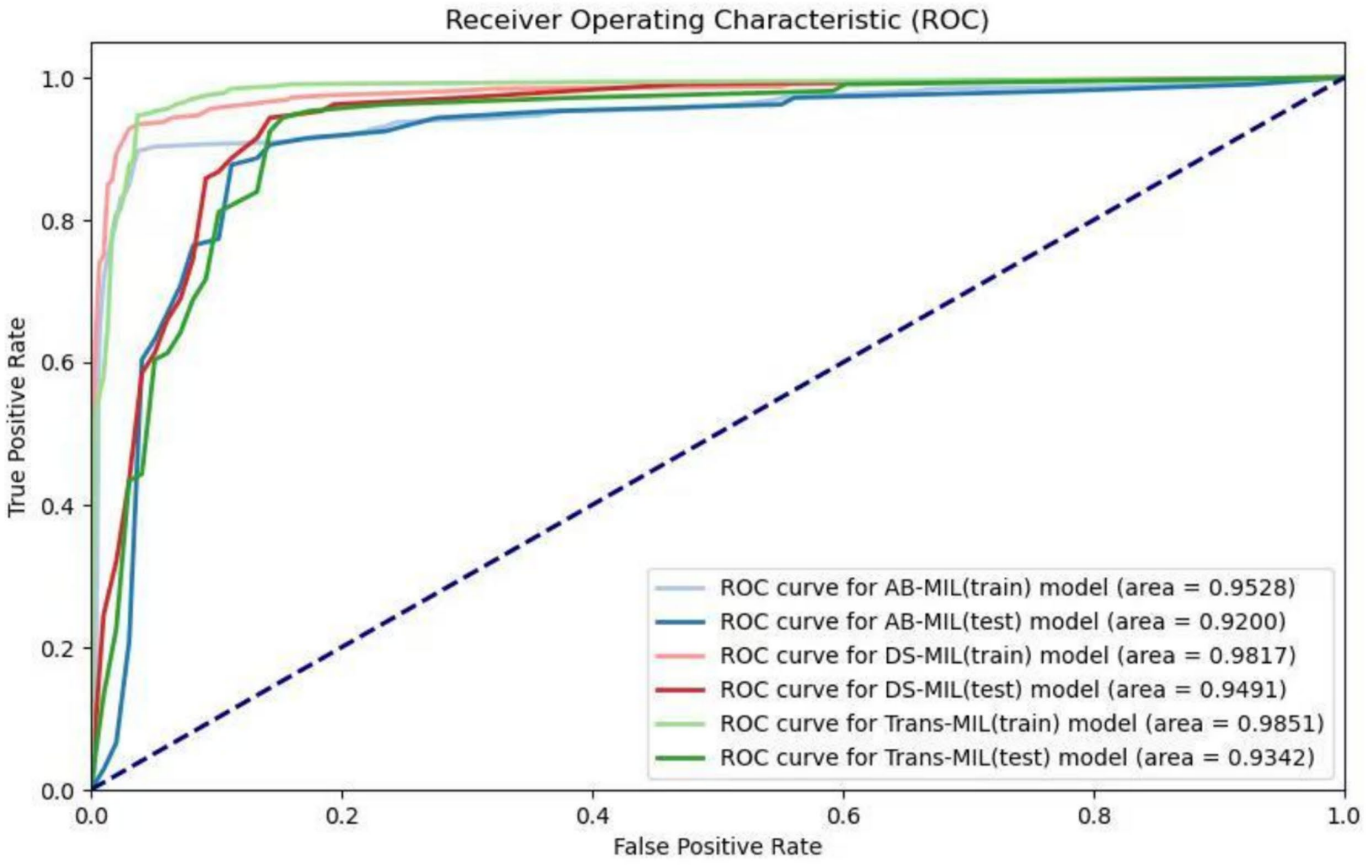
Receiver operating characteristic (ROC) curves of the three models. The lighter colors represent the ROC curves of the AB-MIL, DS-MIL, and Trans-MIL models on the training set, with AUC values of 0.953, 0.982, and 0.985; the darker colors represent the ROC curves of the three models on the testing set, with AUC values of 0.920, 0.949, and 0.934.

As shown in Table 1, the AB-MIL model demonstrated excellent performance metrics, with an accuracy of 88.7%, precision of 88.8%, sensitivity of 87.9%, and specificity of 89.5%. This model demonstrates a false negative rate of 12.1%, a false positive rate of 10.5%, along with an F1-score of 0.883, and an AUC of 0.920. This model outperforms the other two in terms of precision and may be more suitable for application scenarios requiring high certainty. The DS-MIL model has demonstrated exceptional diagnostic capabilities, with the highest accuracy of 89.7% and the highest AUC of 0.949 among the three models. It achieves a precision of 83.7%, sensitivity of 94.3%, and specificity of 86.3%. Additionally, the DS-MIL model exhibits a low false negative rate of 5.7% and a false positive rate of 13.7%, along with an F1-score of 0.888. These findings suggest that the DS-MIL model is not only accurate but also consistently reliable in diagnostic tasks. In contrast, the Trans-MIL model, showing the highest sensitivity of 95.2% in comparison to DS-MIL and AB-MIL, achieves a precision of 80.6%, specificity of 84.3%, and the lowest false negative rate of 4.8%. This is complemented by an F1-score of 0.873 and an AUC of 0.934, highlighting the Trans-MIL model’s effectiveness in accurately identifying *H. pylori*-positive cases, which is crucial for minimizing the rate of missed diagnoses. The comparative efficacy of the models is further illustrated by the ROC curves depicted in Figure 3.

**Table 1:** Performance metrics of three models.

Models	ACC/%	PRE/%	SEN/%	SPE/%	FNR/%	FPR/%	F1-score	AUC
AB-MIL	88.7	88.8	87.9	89.5	12.1	10.5	0.883	0.920
DS-MIL	89.7	83.7	94.3	86.3	5.7	13.7	0.888	0.949
Trans-MIL	88.7	80.6	95.2	84.3	4.8	15.7	0.874	0.934

ACC, accuracy; PRE, precision; SEN, sensitivity; SPE, specificity; FNR, false negative rate; FPR, false positive rate; F1-score: A metric for evaluating the accuracy of a binary classification model.

By conducting the Shapiro–Wilk test, we found that AB-MIL (*p* = 0.0036), DS-MIL (*p* = 0.0086), and Trans-MIL (*p* = 0.0142) exhibited statistically significant differences in their performance, deviating from a normal distribution. Consequently, we employed the Kruskal–Wallis test to compare the metric differences among the models. The results indicated no significant statistical differences in diagnostic performance among the three models (*p* = 0.9614).

### The reliability of DS-MIL can be validated by visual model

**Figure 4:**
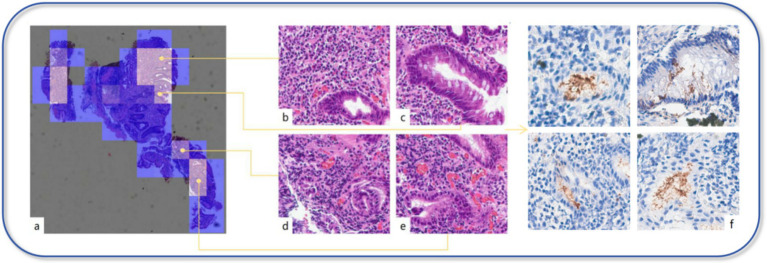
Visual model. **(a)** Each patch’s predicted probability value is mapped onto the heatmap, displaying the importance of each patch or the model’s confidence in its classification through varying colors. **(b–e)** In the heatmap, the brighter the color represents the higher the predicted probability value for that area. Through our review, these areas mostly exhibit significant inflammation, the presence of intestinal metaplasia, dilated and congested interstitial blood vessels, or hemorrhage. **(f)** In the IHC section, the areas that the model focuses on highly indeed show strong positive expression of *H. pylori*.

Although there was no statistically significant difference in diagnostic performance among the three models, DS-MIL achieved the highest AUC and ACC, leading us to select this model for visualization analysis. To elucidate the model’s focal To elucidate the model’s focal points, a heatmap was constructed to graphically represent the attention distribution across different patches within the model, as depicted in Figure 4. Typically, the model’s salient patches, which are the top 50 with the highs predicted probabilities, are selected for further analysis, as these are deemed to be the most significant contributors to the model’s predictive power (17). Upon review by pathologists, it was determined that the majority of the patches, which garnered the model’s attention, contained features such as acute and chronic inflammatory cell infiltration, intestinal metaplasia, and mucosal damage. These observations corroborate the model’s predictive accuracy and reliability in diagnostic applications.

### Diagnostic performance variation among pathologists across different levels

The diagnostic performance of pathologists across different levels has been demonstrated in Tables 2, 3, with all participants being blinded to the outcomes of the IHC. The junior pathologist demonstrated a diagnostic accuracy of 67.0%, a specificity of 45.4%, a sensitivity of 88.9%, a false negative rate of 11.1%, and a false positive rate of 54.5%. The intermediate pathologist displayed a diagnostic accuracy of 73.9%, a specificity of 55.0%, a sensitivity of 92.7%, a false negative rate of 7.3%, and a false positive rate of 45.0%. The senior pathologist demonstrated a diagnostic accuracy of 81.7%, a specificity of 94.5%, a sensitivity of 68.5%, a false negative rate of 31.5%, and a false positive rate of 5.5%. The elevated false positive rates observed among junior and intermediate pathologists underscore an imperative need for enhancements in diagnostic precision. Despite the senior pathologists demonstrating the most impressive diagnostic accuracy, the sensitivity of 68.5% and a false negative rate of 31.5%, suggest that a negative case being misdiagnosed as positive is minimal, a considerable number of actual infections may still remain undetected.

**Table 2:** Average confusion matrix across pathologist levels.

Pathologists’ level	Pathologists’ diagnosis	*H. pylori* IHC		Total
		*H. pylori* (+)	*H. pylori* (−)	
Junior pathologists	*H. pylori* (+)	96	60	156
	*H. pylori* (−)	12	50	62
	Total	108	110	218
Intermediate pathologists	*H. pylori* (+)	101	49	150
	*H. pylori* (−)	8	60	68
	Total	109	109	218
Senior pathologists	*H. pylori* (+)	74	6	80
	*H. pylori* (−)	34	104	138
	Total	108	110	218

Each group consists of 2–4 pathologists, with the confusion matrix based on their average scores.

**Table 3:** Diagnostic evaluation indicators of pathologists.

Pathologists’ level	ACC/%	SEN/%	SPE/%	FNR/%	FPR/%
Junior pathologists	67.0	88.9	45.5	11.1	54.5
Intermediate pathologists	73.9	92.7	55.0	7.3	45.0
Senior pathologists	81.7	68.5	95.3	31.5	5.5
*χ*^2^/*P* (primary vs. intermediate pathologists)	2.477/0.116	0.922/0.377	2.015/0.156	0.922/0.337	2.015/0.156
*χ*^2^/*P* (intermediate vs. senior pathologists)	3.832/0.050	20.257/<0.001	45.419/<0.001^▲^	20.257/<0.001	45.419/<0.001^▲^
*χ*^2^/*P* (primary vs. senior pathologists)	12.303/<0.001^▲^	13.369/<0.001	63.117/<0.001^▲^	13.369/<0.001	63.117/<0.001^▲^

The diagnostic performance of pathologists indicates that there is no statistical significance between junior and intermediate pathologists. In contrast, senior pathologists demonstrate significantly higher evaluation indicators compared to both junior and intermediate pathologists. ^▲^Significance, *p* < 0.05.

### The DS-MIL model outperforms pathologists in comprehensive performance

**Figure 5:**
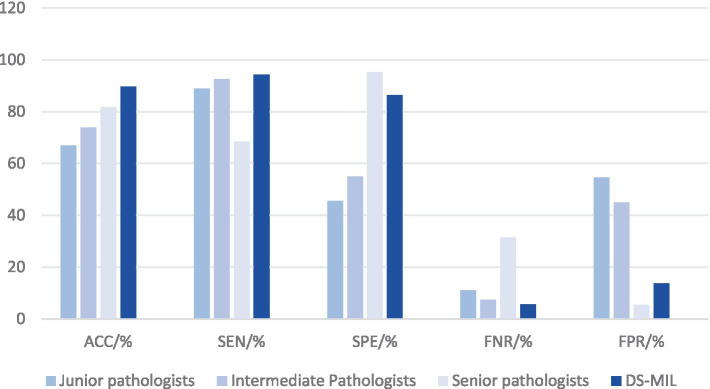
Diagnostic efficacy of DS-MIL and pathologists at all levels. Despite a slightly higher FNR, the DS-MIL’s overall performance is satisfactory, particularly in terms of ACC, SPE, and FPR.

Due to the superior performance of DS-MIL, we compared the DS-MIL model with pathologists, as detailed in Figure 5 and Table 4. The model has significantly surpassed the diagnostic accuracy of junior (*p* < 0.001), intermediate (*p* < 0.001), and even senior pathologists (*p* = 0.019), highlighting its substantial potential in improving the accuracy of diagnosing *H. pylori* infections. Additionally, the DS-MIL model excelled in both specificity and false positive rate, significantly outperforming junior and intermediate pathologists (*p* < 0.001). Furthermore, the DS-MIL model demonstrated superior performance in sensitivity and false negative rate, significantly outperforming senior pathologists (*p* < 0.001). These findings suggest that the DS-MIL model is valuable for enhancing diagnostic accuracy and efficiency, especially in minimizing missed diagnoses.

**Table 4:** Comparison of diagnostic efficacy between DS-MIL and pathologists.

Comparison	ACC	SEN	SPE	FNR	FPR
*χ*^2^/*P* (DS-MIL vs. Junior pathologists)	31.699/<0.001^▲^	1.742/0.187	42.523/<0.001^▲^	1.742/0.187	42.523/<0.001^▲^
*χ*^2^/*P* (DS-MIL vs. intermediate pathologists)	17.577/<0.001^▲^	0.198/0.656	26.945/<0.001^▲^	0.198/0.656	26.945/<0.001^▲^
*χ*^2^/*P* (DS-MIL vs. senior pathologists)	5.529/0.019^▲^	19.944/<0.001^▲^	4.378/0.036	19.944/<0.001^▲^	4.378/0.036

Chi-squared (*χ*^2^) test. ^▲^Significance, *P* < 0.05.

## Discussion

*Helicobacter pylori* is intricately linked with multiple gastric conditions, and its deleterious effects are gaining increasing recognition. It is estimated that approximately 5.5% of all cancers globally are related to *H. pylori* infection, accounting for 25% of all infection-related malignancies (2, 6, 18). However, due to the microscale of *H. pylori* and the interference from gastric mucosal secretions and impurities within the gastric pits that impede visualization, the diagnostic process is exceedingly labor-intensive, and the diagnostic quality is also suboptimal. Consequently, we aimed to develop an AI-assisted diagnostic tool designed to enhance diagnostic precision and alleviate the workload of pathologists.

Liscia et al. (19) developed a model for detecting *H. pylori* in W-S stained images, with an AUC of 0.93. Klein et al. (20) conducted a similar study, in which they achieved an AUC of 0.92 for identifying *H. pylori* in Giemsa-stained sections, but it fell to 0.81 with H&E staining. Lin et al. (21) developed a two-tier deep-learning-based model to diagnose *H. pylori* infection with pathologist-level accuracy on H&E-stained sections. Based on the morphological alterations resulting from *H. pylori* infection, Franklin et al.’s (22) CNN model matches gastrointestinal pathologists in accuracy for distinguishing *H. pylori* gastritis from autoimmune gastritis. The team also trained a model to distinguish between normal gastric mucosa, *H. pylori*-associated gastritis, and reactive gastropathy (23), achieving an AUC of 100% for recognizing *H. pylori*-associated gastritis. This demonstrates the feasibility of predicting the presence or absence of *H. pylori* infection predicated on morphological features.

Compared to some other studies, the three models in our study are capable of directly detecting *H. pylori* infection on H&E-stained sections without additional IHC or special staining. We utilized a substantial dataset comprising 817 cases with a total of 1,020 H&E-stained images. The scale of this dataset is particularly remarkable compared to similar studies, providing a solid foundation for our models training. Moreover, we meticulously designed the experimental procedure, allocating 60% of these cases for training and optimizing models, ensuring that the model can learn sufficient features and patterns from the rich data, thereby enhancing its generalization capabilities and predictive accuracy. As a result, our three models outperformed most comparable studies in terms of AUC performance.

Multi-Instance Learning (MIL) is a form of weakly supervised learning that addresses the unique challenge of data representation and classification. Unlike conventional supervised learning techniques, where each training instance is linked to a specific label, MIL operates under the premise that data is organized into discrete ‘bags’, with labels assigned only at the bag level, rather than to individual instances within the bags. This method is particularly advantageous in scenarios where obtaining labels for each instance is impractical or infeasible, reducing the burden of manual annotation while capitalizing on the benefits of weak supervision. In this study, we constructed three MIL models, all of which exhibit distinctive strengths. In our study, although there was no statistically significant difference in performance among the three models, they each demonstrated distinct strengths and focuses. The AB-MIL demonstrated a balanced performance, achieving an accuracy of 88.7% and a precision of 88.8%. Nonetheless, it did not show a significant advantage in any particular metric, and the model’s false negative rate (12.1%) and false positive rate (10.5%) were relatively high. The Trans-MIL demonstrated the best performance in sensitivity (95.2%) and false negativity rate (4.8%), showing a typical capability to accurately identify *H. pylori*-positive cases ensures that the maximum number of truly infected patients are detected. However, this led to a decrease in specificity (84.3%) and an increase in the false positivity rate (15.7%), resulting in a higher risk of misdiagnosis. In contrast, the DS-MIL stood out with its superior accuracy (89.7%) and AUC values (0.949), becoming the top choice for comprehensive diagnostic capabilities and the most suitable MIL model for *H. pylori* detection, offering a reliable and efficient solution to enhance diagnostic accuracy in clinical application.

Upon analysis of the diagnostic outcomes from pathologists of varying levels of expertise, it is evident that both junior and intermediate pathologists exhibit increased FPRs, with rates of 54.5 and 45.0%, respectively. This trend suggests a proclivity for these pathologists to erroneously categorize *H. pylori*-negative cases as *H. pylori*-positive, potentially due to the impact of debris within the gastric pits. In contrast, the FPRs are not elevated among senior pathologists. This may be attributed to their extensive diagnostic experience, which renders them less susceptible to misinterpretation by extraneous factors. However, it is possible that the relatively high FNR of the senior pathologist may result in the inadvertent omission of some authentic *H. pylori*-positive cases. Our three models, particularly the DS-MIL, have demonstrated diagnostic accuracy and sensitivity that exceeds that of senior pathologists while achieving specificity higher than that of junior and intermediate pathologists. Furthermore, there is a notable reduction in FNRs, which is of considerable importance in enhancing diagnostic precision and minimizing the occurrence of missed diagnosis. Additionally, the evident advantages of AI models in processing large volumes of data allow them to analyze a vast number of WSI in a relatively short period. This capability markedly enhances diagnostic efficiency and alleviates the workload of pathologists. In summary, when compared with pathologists, the advantages of DS-MIL remain significant.

Although our three models have demonstrated excellent diagnostic performance, there are still several issues that need to be addressed prior to their clinical application in pathological practice. First, the data we used for developing this AI-assisted pathology diagnostic system was sourced solely from a single center. Although the predictive performance of the three models in the test set is already satisfactory, further validation with multi-center datasets is still needed. Secondly, in accordance with the expert consensus on the pathological histology of *H. pylori* infection, the severity of *H. pylori* infection is categorized into four grades: none, mild, moderate, and severe, and *H. pylori* can be further classified into two molecular subtypes, CagA and VacA (24). To date, our models are capable of predicting the presence of *H. pylori* infection but are not yet equipped to assess the severity of infection or to identify molecular subtypes, which remains a target for our ongoing research. Lastly, this study did not record the medication history of patients within 1–2 months prior to gastric mucosal biopsy, especially the use of non-steroidal anti-inflammatory drugs, proton pump inhibitors, and antibiotics. In further research, we hope to integrate clinical symptoms, medication history, and other clinical data, as well as images and diagnoses from gastrointestinal endoscopy, to construct a multimodal AI diagnostic system that can integrate multidisciplinary information and provide comprehensive diagnoses.

In conclusion, the findings of our study underscore that the three models exhibit superior performance compared to traditional *H. pylori* detection methods across a range of evaluative metrics, including diagnostic accuracy, precision, sensitivity, and specificity. Among them, DS-MIL has become the preferred choice due to its optimal diagnostic performance. It has the potential to replace pathologists in detecting *H. pylori* infections in the future, significantly improving the accuracy and efficiency of diagnosis. However, currently the predictions generated by AI models must be subjected to review and confirmation by pathologists prior to the issuance of pathology reports.

## Data Availability

The original contributions presented in the study are included in the article/supplementary material, further inquiries can be directed to the corresponding authors.
